# Supplementary figures and images for: A connection between the ribosome and two S. pombe tRNA modification mutants subject to rapid tRNA decay
Source: PLoS Genet. 2024 Jan 31;20(1):e1011146. doi: 10.1371/journal.pgen.1011146 (PMC10861057; doi:10.1371/journal.pgen.1011146)

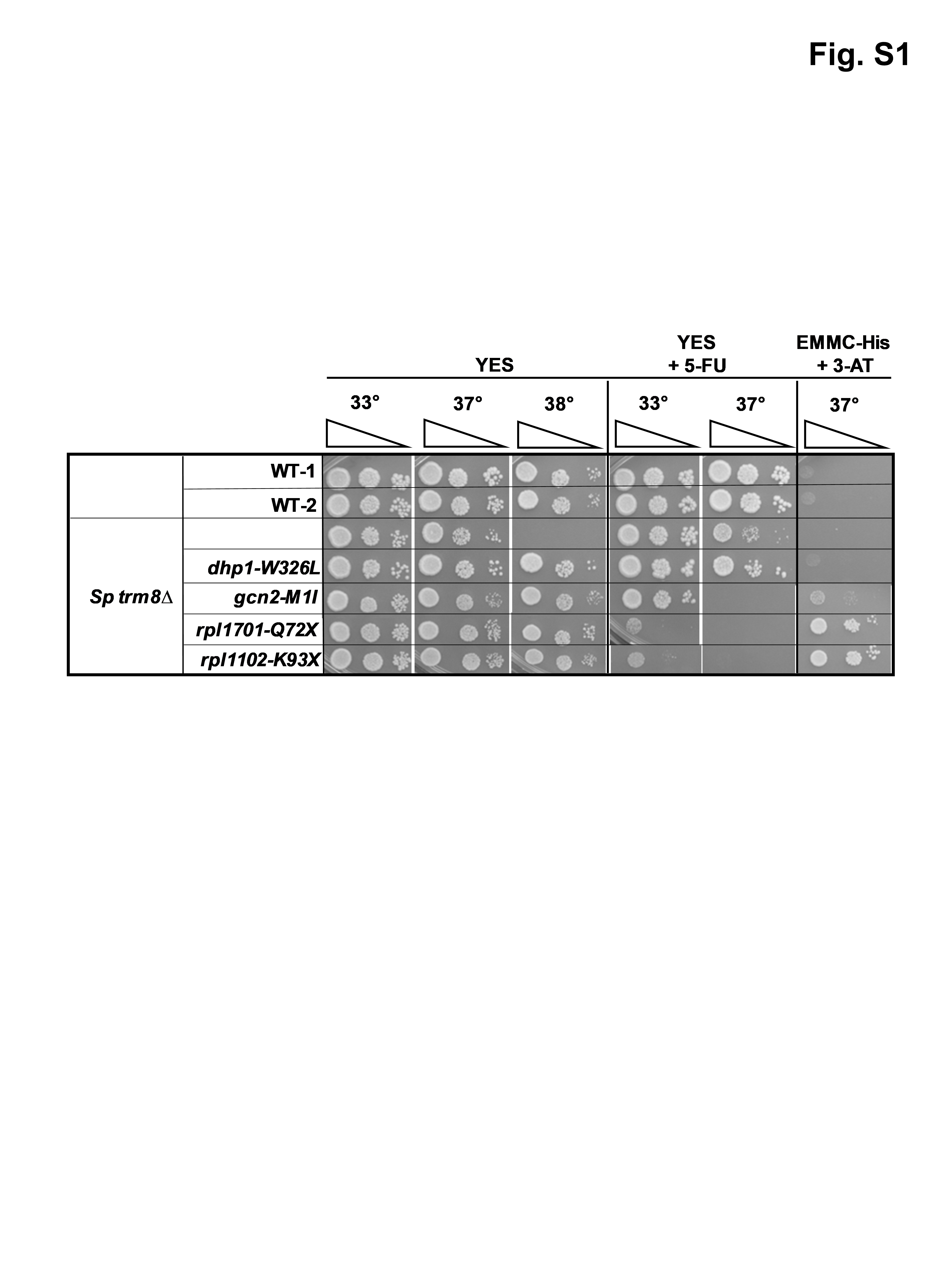

Supplement: S1 Fig — S. pombe trm8Δ mutants and suppressors as indicated were grown overnight in YES media at 30°C and analyzed for growth on plates as described in Fig 1A. (TIF) [file pgen.1011146.s001.TIF]

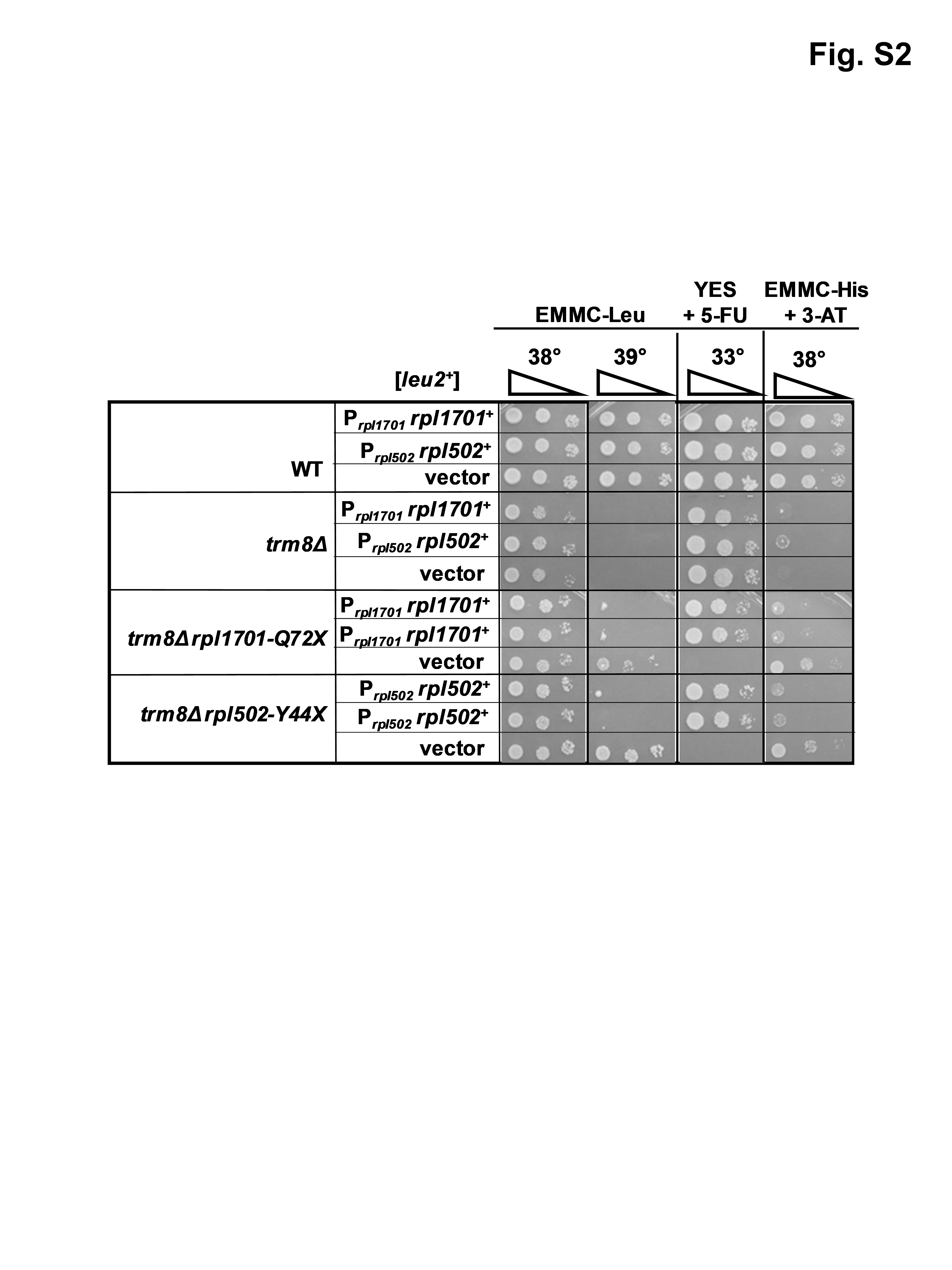

Supplement: S2 Fig — S. pombe trm8Δ rpl502-Y44X and trm8Δ rpl1701-Q72X mutants were transformed with a [leu2+ rpl502+] or [leu2+ rpl1701+] plasmid respectively, or a [leu2+] vector control, and transformants were grown overnight in EMMC-Leu media at 30°C and analyzed for growth, as in Fig 1A, on plates containing EMMC-Leu, YES with 5-FU (30 μg/ml), and EMMC-His with 10 mM 3-AT. (TIF) [file pgen.1011146.s002.TIF]

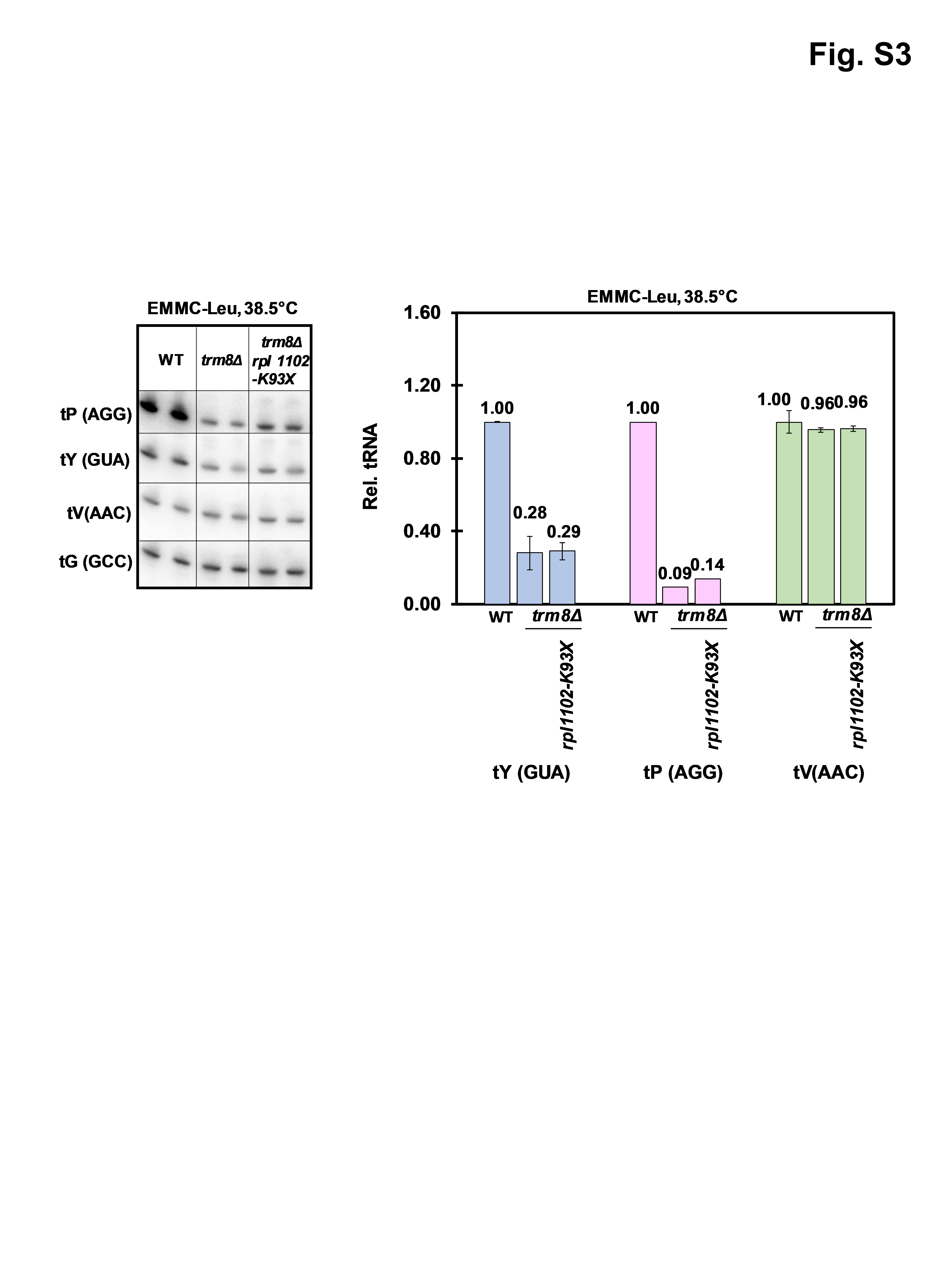

Supplement: S3 Fig — Strains were transformed with a leu2+ vector and then grown in EMMC-Leu media at 30°C and shifted to 38.5°C. Growth was monitored for 10 hours before harvest, and then bulk RNA was isolated and analyzed by northern blot analysis as described in Materials and Methods, and tRNA levels were quantified as described in Fig 1C. Note that for this experiment biological replicates were compared, rather than triplicates. (TIF) [file pgen.1011146.s003.TIF]

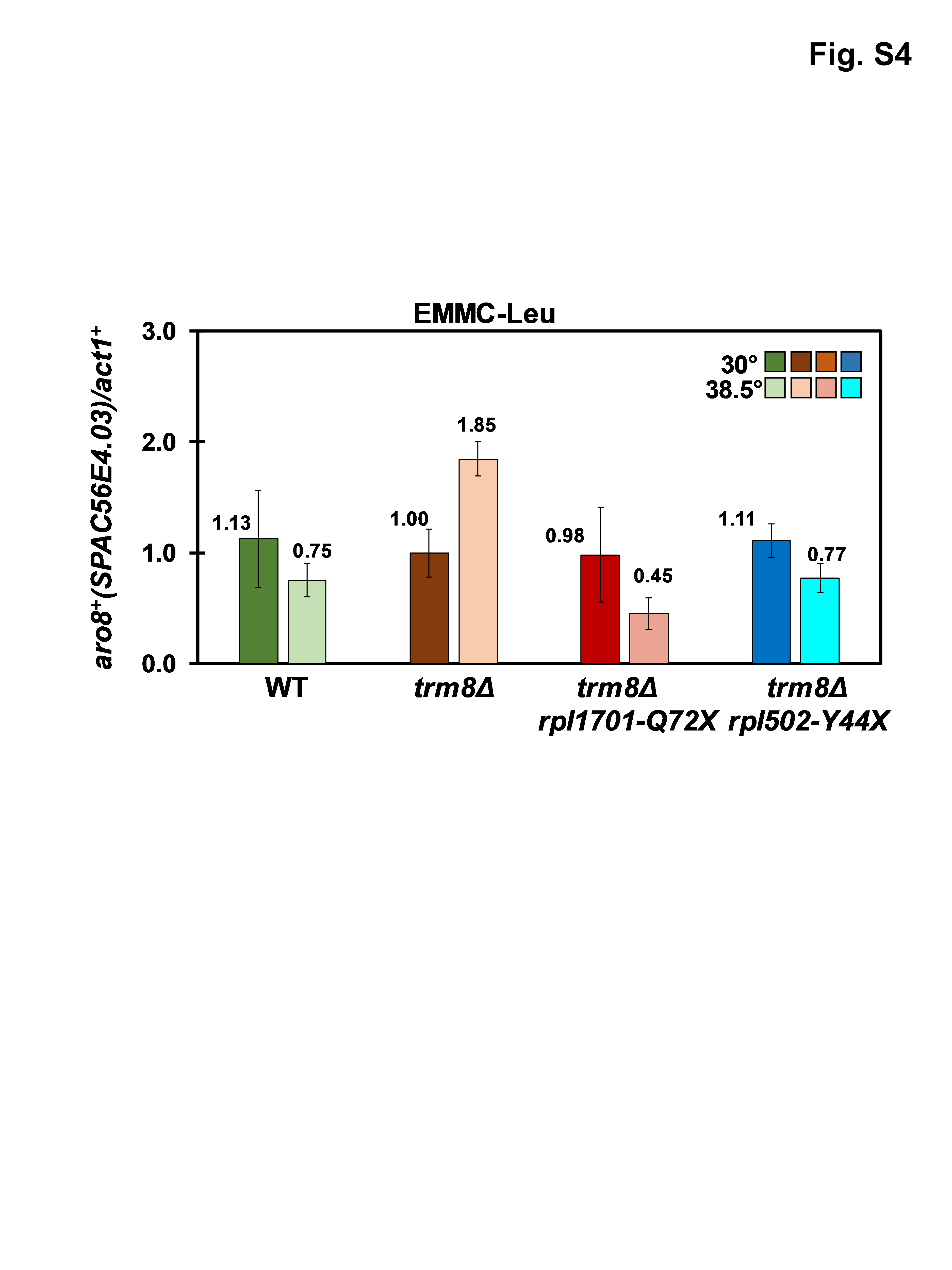

Supplement: S4 Fig — Bulk RNA from the experiment in Fig 1B was used to analyze aro8+ mRNA levels, normalized as described in Fig 1D. (TIF) [file pgen.1011146.s004.TIF]

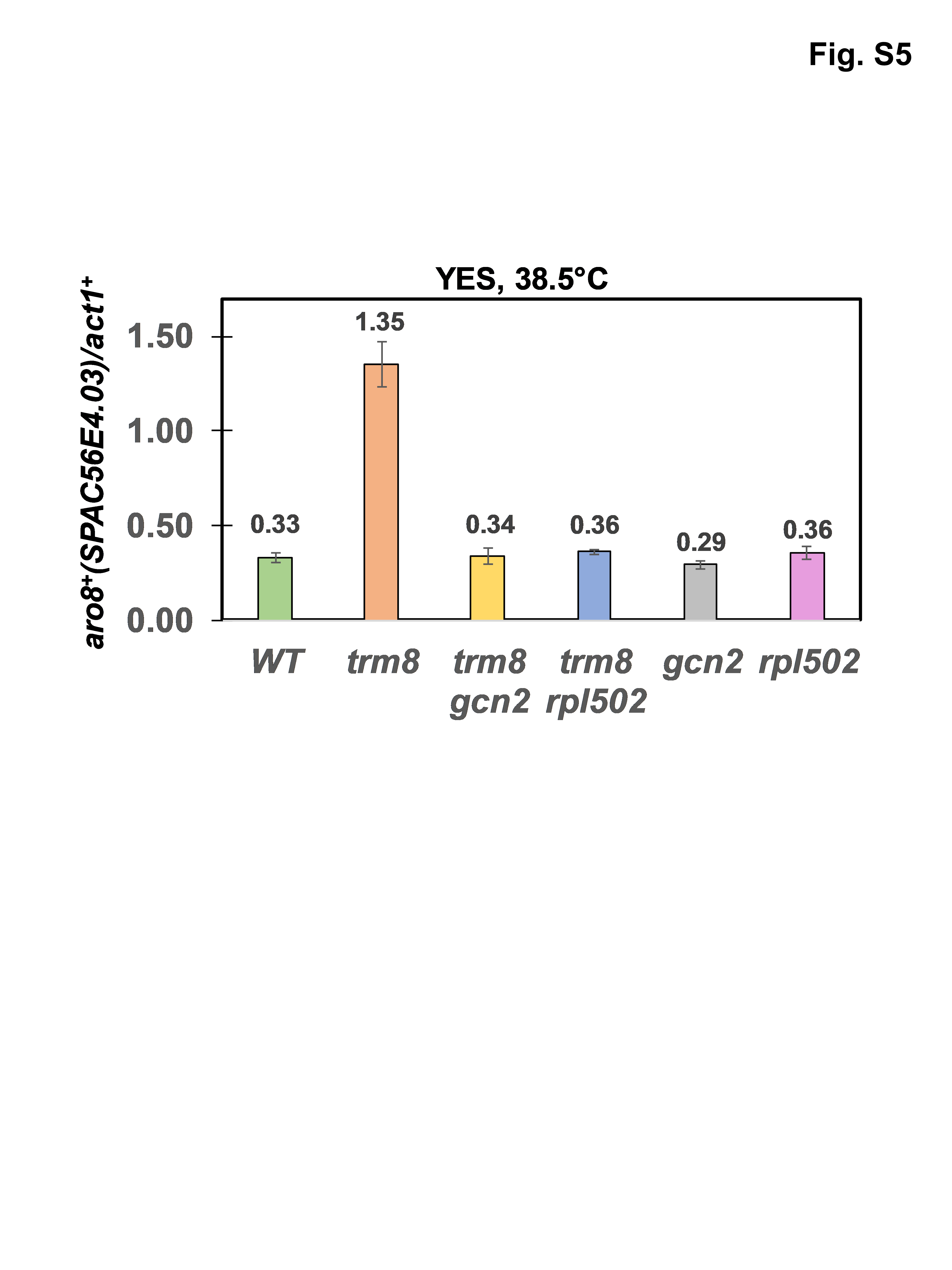

Supplement: S5 Fig — Bulk RNA from the experiment in Fig 2B was used to analyze aro8+ mRNA levels, as described in Fig 1D. (TIF) [file pgen.1011146.s005.TIF]

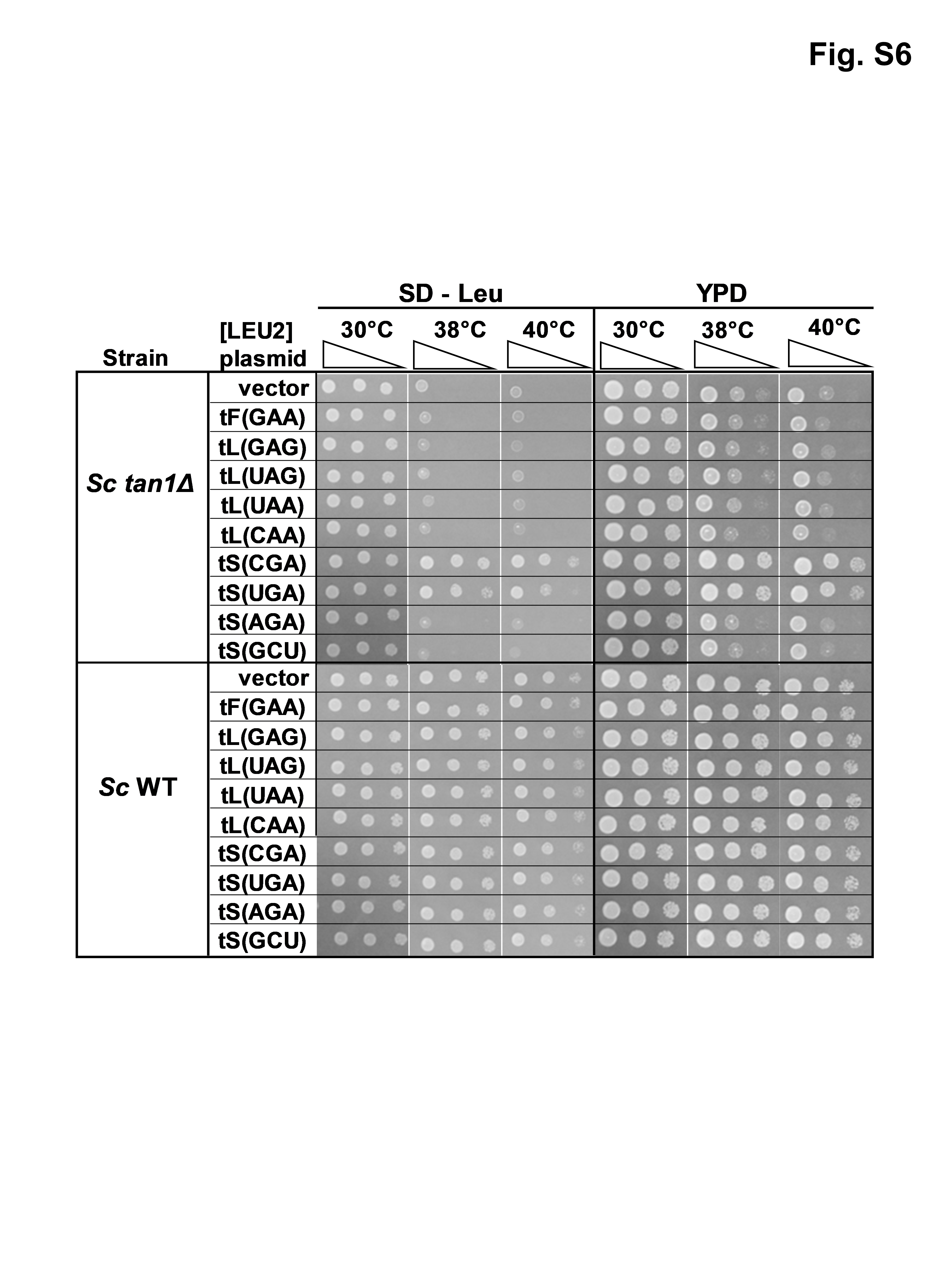

Supplement: S6 Fig — S. cerevisiae WT and tan1Δ mutants were transformed with a [LEU2+] plasmid expressing tRNAs as indicated, or a vector control, and transformants were grown overnight in SD—Leu media at 30°C and analyzed for growth as in Fig 1A, on plates containing SD–Leu or YPD media. (TIF) [file pgen.1011146.s006.TIF]

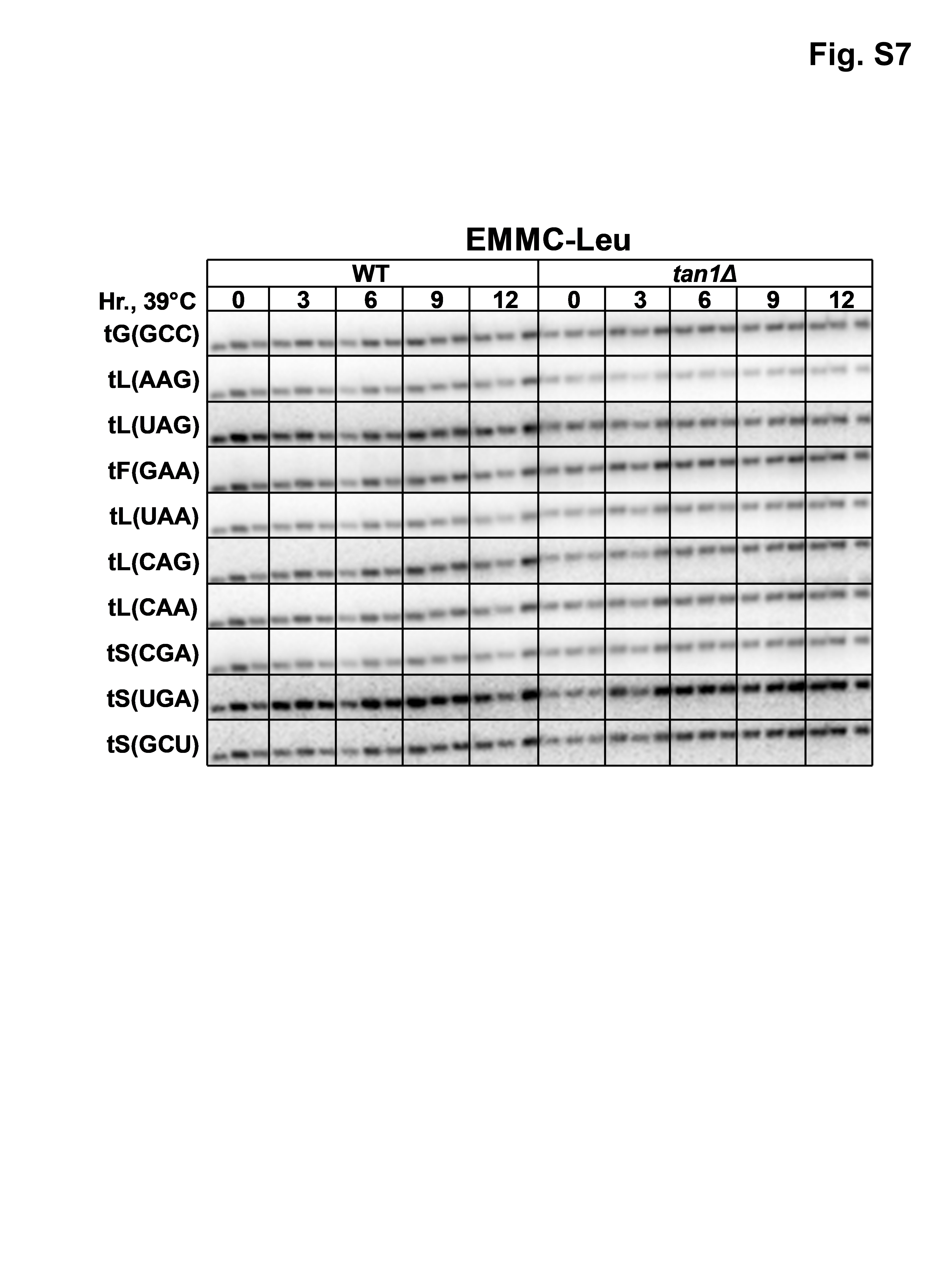

Supplement: S7 Fig — S. pombe WT and tan1Δ mutants were transformed with a [leu2+] plasmid, and transformants were grown in EMMC-Leu media to mid-log phase, diluted and shifted to 39°C and grown for 12 hours, and RNA was isolated at 0, 3, 6, 9, and 12 hours and analyzed by Northern blot as described in Materials and Methods, with the indicated probes. (TIF) [file pgen.1011146.s007.TIF]

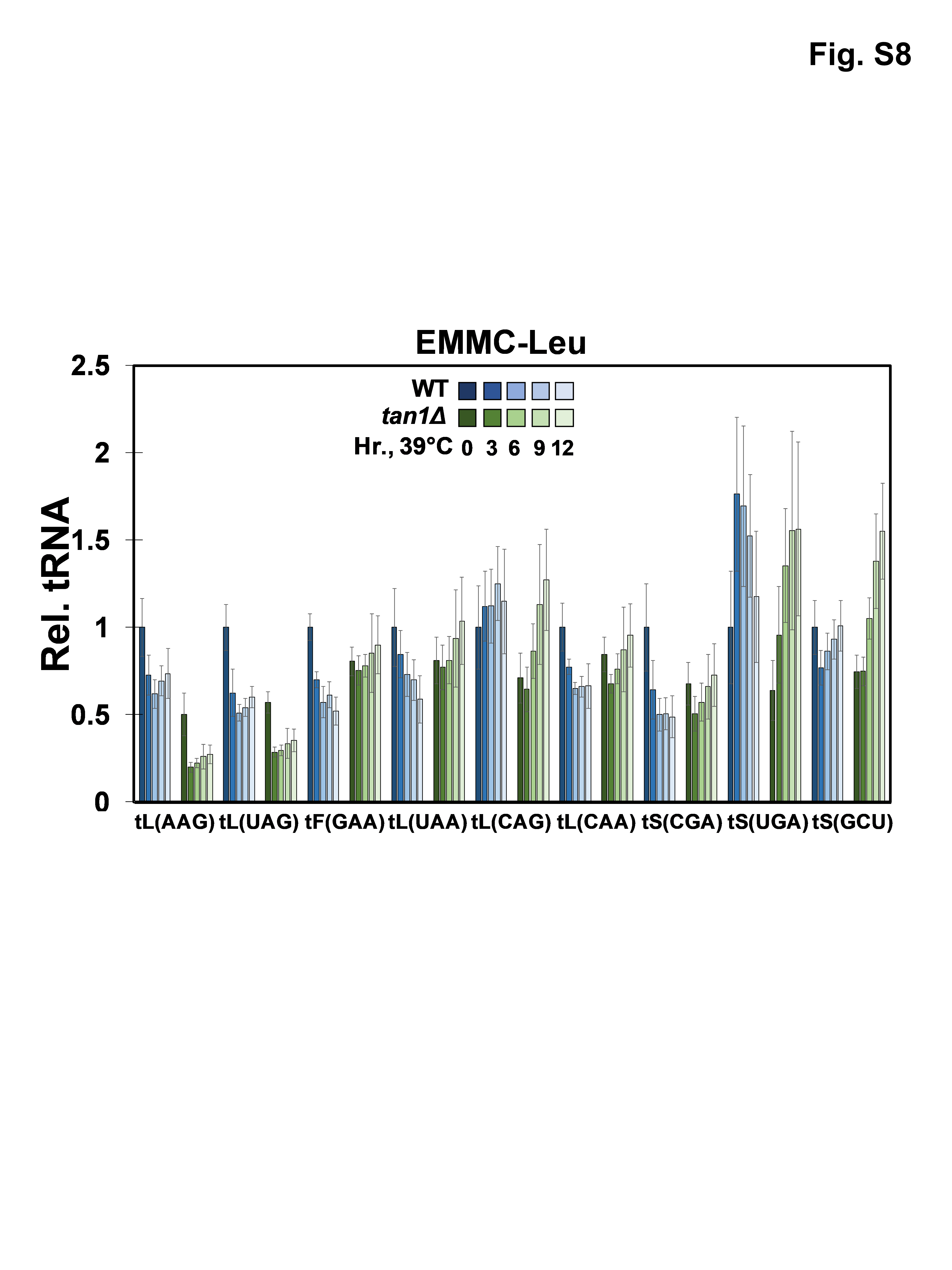

Supplement: S8 Fig — The Northern in S7 Fig was quantified as described in Fig 1C. Note that the data in Fig 4C is from this quantification. shades of blue, WT strains analyzed at 0 (darkest) through 12 hours (lightest); shades of green, tan1Δ strains. (TIF) [file pgen.1011146.s008.TIF]

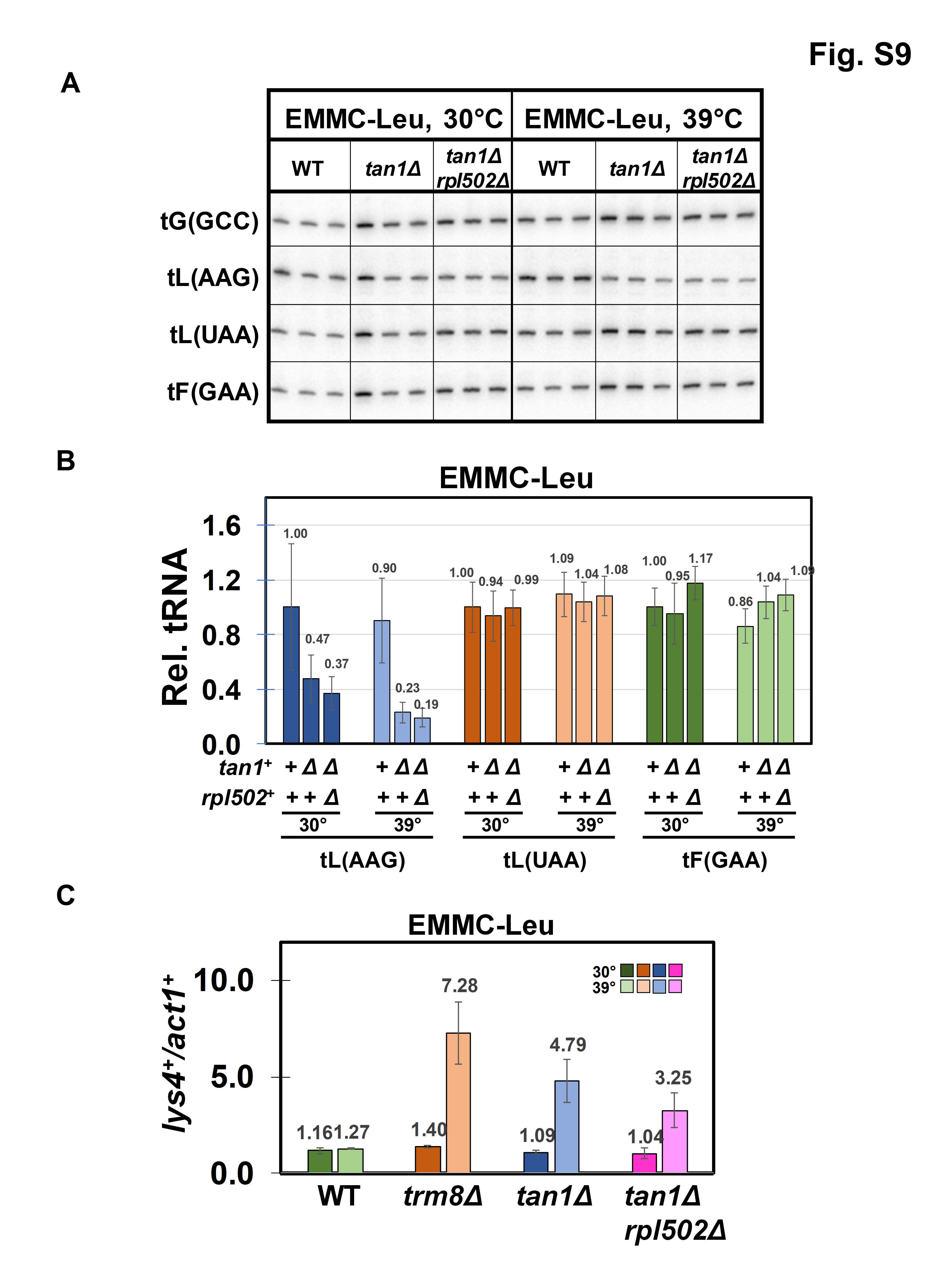

Supplement: S9 Fig — (A). An S. pombe tan1Δ rpl502Δ suppressor does not restore the tRNALeu(AAG) decay observed in tan1Δ mutants in SD–Leu media. S. pombe WT, tan1Δ, and tan1Δ rpl502Δ mutants were transformed with a [leu2+] plasmid, and transformants were grown in EMMC—Leu media to mid-log phase, diluted into fresh media at 30°C and 39°C and grown for 9 hours, and then bulk RNA was analyzed for tRNA levels by northern blot analysis as described in Materials and Methods, with the indicated probes. (B). Quantification of tRNA levels of WT, tan1Δ, and tan1Δ rpl502Δ mutants. (C). Analysis of GAAC activation. Bulk RNA from the growth in S9A Fig was analyzed for GAAC activation as described in Fig 1D. (TIF) [file pgen.1011146.s009.TIF]

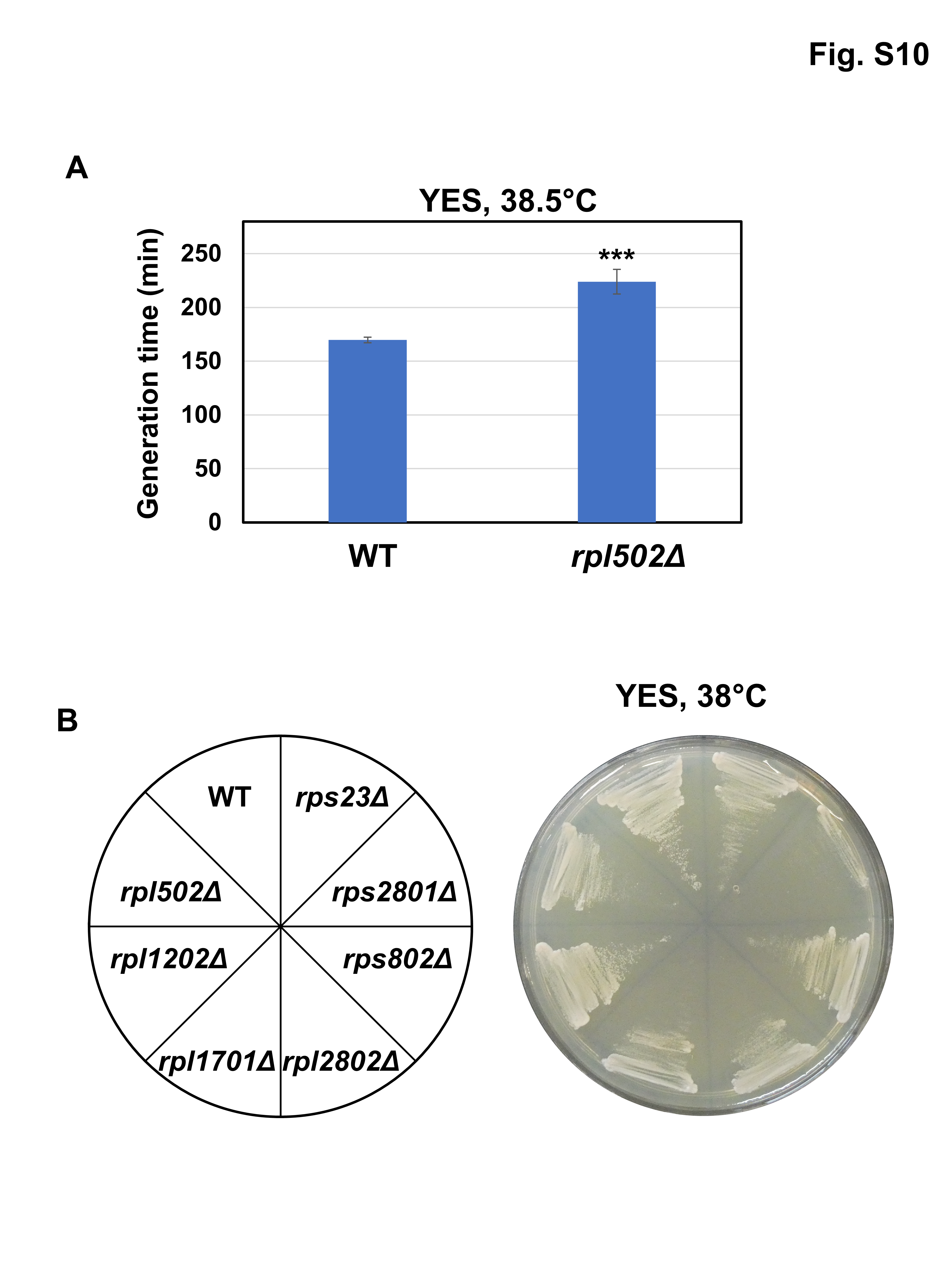

Supplement: S10 Fig — S. pombe WT and rpl502Δ strains were grown in liquid YES media at 38.5°C and growth was monitored every hour for 8 hours to measure the growth rate. (B). Each of several S. pombe rplΔ and rpsΔ strains have growth defects. S. pombe WT, rplΔ, and rpsΔ strains as indicated were streaked on plates containing YES media, and incubated at 38°C for 2 days. (TIF) [file pgen.1011146.s010.TIF]
