# Supplementary material for: A connection between the ribosome and two S. pombe tRNA modification mutants subject to rapid tRNA decay
Source: PLoS Genet. 2024 Jan 31;20(1):e1011146. doi: 10.1371/journal.pgen.1011146 (PMC10861057; doi:10.1371/journal.pgen.1011146)
Supplement: S1 Table — (DOCX) [file pgen.1011146.s011.docx]

**Table S1. Parameters for growth curves and derived generation times of *S. pombe* WT, *rplΔ*, and *rpsΔ* strains**

| **Strain** | **Genotype** | **Exponent** | **Generation Time (min.)** | **R^2^** |
| --- | --- | --- | --- | --- |
| YAH927a-1 | WT | 0.253 | 165 | 0.996 |
| YAH927a-2 | WT | 0.255 | 163 | 0.998 |
| YAH927a-3 | WT | 0.251 | 166 | 0.994 |
| YTD604-1A | WT *rpl502Δ* | 0.187 | 222 | 0.998 |
| YTD604-1B | WT *rpl502Δ* | 0.185 | 225 | 0.997 |
| YTD604-2 | WT *rpl502Δ* | 0.177 | 236 | 0.979 |
| YAH957-1A | WT *rpl1202Δ* | 0.215 | 193 | 0.998 |
| YAH957-1B | WT *rpl1202Δ* | 0.212 | 197 | 0.998 |
| YAH957-2 | WT *rpl1202Δ* | 0.215 | 193 | 0.991 |
| YAH964-1A | WT *rpl1701Δ* | 0.174 | 239 | 0.985 |
| YAH964-1B | WT *rpl1701Δ* | 0.179 | 233 | 0.988 |
| YAH964-2 | WT *rpl1701Δ* | 0.170 | 244 | 0.987 |
| YAH958-1A | WT *rpl2802Δ* | 0.176 | 237 | 0.959 |
| YAH958-1B | WT *rpl2802Δ* | 0.183 | 228 | 0.985 |
| YAH958-2 | WT *rpl2802Δ* | 0.175 | 238 | 0.924 |
| YAH967-1A | WT *rps802Δ* | 0.227 | 183 | 0.999 |
| YAH967-1B | WT *rps802Δ* | 0.227 | 183 | 0.990 |
| YAH967-2 | WT *rps802Δ* | 0.224 | 186 | 0.996 |
| YAH952-2A | WT *rps2801Δ* | 0.191 | 217 | 0.991 |
| YAH952-2B | WT *rps2801Δ* | 0.190 | 219 | 0.988 |
| YAH952-3 | WT *rps2801Δ* | 0.177 | 235 | 0.996 |
| YAH965-1A | WT *rps23Δ* | 0.209 | 199 | 0.998 |
| YAH965-1B | WT *rps23Δ* | 0.193 | 215 | 0.991 |
| YAH965-2 | WT *rps23Δ* | 0.192 | 216 | 0.996 |
