# Supplementary material for: A connection between the ribosome and two S. pombe tRNA modification mutants subject to rapid tRNA decay
Source: PLoS Genet. 2024 Jan 31;20(1):e1011146. doi: 10.1371/journal.pgen.1011146 (PMC10861057; doi:10.1371/journal.pgen.1011146)
Supplement: S2 Table — (DOCX) [file pgen.1011146.s012.docx]

**Table S2. *S. pombe* strains used in this study**

| Strain | Parent | Genotype | Source |
| --- | --- | --- | --- |
| YTD 367-3 |  | WT | (1) |
| YTD 369-3 |  | *trm8Δ::kanR* (original) | (1) |
| YTD 556-4 | YTD 367-3 | WT *trm8Δ::kanR* (re-made) | (1) |
| YTD 408-1A | YTD 369-3 | *trm8Δ::kanR dhp1-W326L* | (1) |
| YTD 408-1E | YTD 369-3 | *trm8Δ::kanR gcn2-M1I* | (1) |
| YTD 375-3B | YTD 369-3 | *trm8Δ::kanR rpl502-Y44X* | this study |
| YTD 375-4H | YTD 369-3 | *trm8Δ::kanR rpl1102-K93X* | this study |
| YTD 375-4A | YTD 369-3 | *trm8Δ::kanR rpl1502-X201E* | this study |
| YTD 408-2G | YTD 369-3 | *trm8Δ::kanR rpl1701-Q72X* | this study |
| YTD 606 | YTD 556-4 | *trm8Δ::kanR rpl502Δ::HygR* | this study |
| YAH 960 | YTD 556-4 | *trm8Δ::kanR rpl1202Δ::HygR* | this study |
| YAH 954 | YTD 556-4 | *trm8Δ::kanR rpl1601Δ::HygR* | this study |
| YAH 969 | YTD 556-4 | *trm8Δ::kanR rpl1701Δ::HygR* | this study |
| YAH 961 | YTD 556-4 | *trm8Δ::kanR rpl2802Δ::HygR* | this study |
| YAH 972 | YTD 556-4 | *trm8Δ::kanR rps802Δ::HygR* | this study |
| YAH 955 | YTD 556-4 | *trm8Δ::kanR rps2801Δ::HygR* | this study |
| YTD 604 | YAH 367-3 | WT *rpl502Δ::HygR* | this study |
| YAH 957 | YAH 367-3 | WT *rpl1202Δ::HygR* | this study |
| YAH 951 | YAH 367-3 | WT *rpl1601Δ::HygR* | this study |
| YAH 964 | YAH 367-3 | WT *rpl1701Δ::HygR* | this study |
| YAH 958 | YAH 367-3 | WT *rpl2802Δ::HygR* | this study |
| YAH 967 | YAH 367-3 | WT *rps802Δ::HygR* | this study |
| YAH 952 | YAH 367-3 | WT *rps2801Δ::HygR* | this study |
| YNV 39-1 | YAH 367-3 | WT *tan1Δ::kanR* (re-made) | this study |
| YEM 71 | YNV 39-1 | *tan1Δ::kanR dhp1-I196T* | this study |
| YEM 67 | YNV 39-1 | *tan1Δ::kanR dhp1-P203L* | this study |
| YEM 46 | YNV 39-1 | *tan1Δ::kanR dhp1-P677S* | this study |
| YEM 69 | YNV 39-1 | *tan1Δ::kanR rpl1101-N7fs* | this study |
| YEM 54 | YNV 39-1 | *tan1Δ::kanR rpl1102-R133fs* | this study |
| YEM 61 | YNV 39-1 | *tan1Δ::kanR rpl1502-R67_R68_InsVR* | this study |
| YEM 43 | YNV 39-1 | *tan1Δ::kanR rpl1701-T35fs* | this study |
| YNV 57-1F | YNV 39-1 | *tan1Δ::kanR rpl2802-G113fs* | this study |
| YEM 65 | YNV 39-1 | *tan1Δ::kanR rpl3001-N80fs* | this study |
| YEM 45 | YNV 39-1 | *tan1Δ::kanR grn1-N37fs* | this study |
| YEM 55 | YNV 39-1 | *tan1Δ::kanR grn1-N37fs* | this study |
| YAH 1006 | YNV 39-1 | *tan1Δ::kanR rpl502Δ::HygR* | this study |
| YAH 1015 | YNV39-1 | *tan1Δ::kanR rpl1701Δ::HygR* | this study |
| YAH 1030 | YNV 39-1 | *tan1Δ::kanR rpl2802Δ::HygR* | this study |
| YAH 1013 | YNV39-1 | *tan1Δ::kanR rps23Δ::HygR* | this study |
| YAH 965 | YAH 367-3 | WT *rps23Δ::HygR* | this study |
| YTD 586 | YTD 367-3 | WT *gcn2Δ::HygR* | (1) |
| YAH 1005 | YNV 39-1 | *tan1Δ::kanR gcn2Δ::HygR* | this study |

**References**

1. De Zoysa T, Phizicky EM. Hypomodified tRNA in evolutionarily distant yeasts can trigger rapid tRNA decay to activate the general amino acid control response, but with different consequences. PLoS Genet. 2020;16(8):e1008893.
