# Supplementary material for: A connection between the ribosome and two S. pombe tRNA modification mutants subject to rapid tRNA decay
Source: PLoS Genet. 2024 Jan 31;20(1):e1011146. doi: 10.1371/journal.pgen.1011146 (PMC10861057; doi:10.1371/journal.pgen.1011146)
Supplement: S3 Table — (DOCX) [file pgen.1011146.s013.docx]

**Table S3. *S. cerevisiae* strains used in this study**

| Strain | Parent | Genotype | Source |
| --- | --- | --- | --- |
| ISC 539 | AA 527 | BY 4741 *trm8Δ*::*natMX trm4Δ*::*URA3* | (1) |
| YAH894 | BY4741 | BY4741 *HIS3^+^* | This study |
| YAH895 | ISC 539 | *trm8Δ*::*natMX trm4Δ*::*URA3 HI3^+^* | This study |
| YAH 934 | YAH 894 | *HIS3^+^ rpl1AΔ*::*kanMX* | This study |
| YAH 936 | YAH 894 | *HIS3^+^ rpl11BΔ*::*kanMX* | This study |
| YAH 937 | YAH 894 | *HIS3^+^ rpl17BΔ*::*kanMX* | This study |
| YAH 938 | YAH 894 | *HIS3^+^ rpl33BΔ*::*kanMX* | This study |
| YAH 939 | YAH 894 | *HIS3^+^ rps0AΔ*::*kanMX* | This study |
| YAH 940 | YAH 894 | *HIS3^+^ rps22AΔ*::*kanMX* | This study |
| YAH 941 | YAH 894 | *HIS3^+^ rps28AΔ*::*kanMX* | This study |
| YAH 942 | YAH 895 | *trm8Δ*::*natMX trm4Δ*::*URA3 HI3^+^ rpl1AΔ::KanMX* | This study |
| YAH 944 | YAH 895 | *trm8Δ*::*natMX trm4Δ*::*URA3 HI3^+^ rpl11BΔ::KanMX* | This study |
| YAH 945 | YAH 895 | *trm8Δ*::*natMX trm4Δ*::*URA3 HI3^+^ rpl17BΔ::KanMX* | This study |
| YAH 946 | YAH 895 | *trm8Δ*::*natMX trm4Δ*::*URA3 HI3^+^ rpl33BΔ::KanMX* | This study |
| YAH 947 | YAH 895 | *trm8Δ*::*natMX trm4Δ*::*URA3 HI3^+^ rps0Δ::KanMX* | This study |
| YAH 948 | YAH 895 | *trm8Δ*::*natMX trm4Δ*::*URA3 HI3^+^ rps22AΔ::KanMX* | This study |
| YAH 949 | YAH 895 | *trm8Δ*::*natMX trm4Δ*::*URA3 HI3^+^ rps28AΔ::KanMX* | This study |

**References**

1. Alexandrov A, Chernyakov I, Gu W, Hiley SL, Hughes TR, Grayhack EJ, et al. Rapid tRNA decay can result from lack of nonessential modifications. Mol. Cell. 2006;21(1):87-96.
