# Supplementary material for: A connection between the ribosome and two S. pombe tRNA modification mutants subject to rapid tRNA decay
Source: PLoS Genet. 2024 Jan 31;20(1):e1011146. doi: 10.1371/journal.pgen.1011146 (PMC10861057; doi:10.1371/journal.pgen.1011146)
Supplement: S4 Table — (DOCX) [file pgen.1011146.s014.docx]

**Table S4. Plasmids used in this study**

| Plasmid | Parent | Description | Source |
| --- | --- | --- | --- |
| pREP3X |  | *[LEU2* P*_nmt1_]* | (1) |
| AB 553-1 | pREP3X | *[LEU2* with a NotI site] | (1) |
| EAH 282-1 | AB 553-1 | *[LEU2* P*_rpl1701_ rpl1701^+^]* | This study |
| EAH 284-1 | AB 553-1 | *[LEU2* P*_rpl502_ rpl502^+^]* | This study |
| ESMO 2-1 | AB 553-2 | *[LEU2 tL(UAG)]* | This study |
| ESMO 14-2 | AB 553-1 | *[LEU2 tL(AAG)]* | This study |
| ESMO 8 | AB 553-2 | *[LEU2 tL(AAG) tL(UAG)]* | This study |

**References**

1. De Zoysa T, Phizicky EM. Hypomodified tRNA in evolutionarily distant yeasts can trigger rapid tRNA decay to activate the general amino acid control response, but with different consequences. PLoS Genet. 2020;16(8):e1008893.
