# Supplementary material for: A connection between the ribosome and two S. pombe tRNA modification mutants subject to rapid tRNA decay
Source: PLoS Genet. 2024 Jan 31;20(1):e1011146. doi: 10.1371/journal.pgen.1011146 (PMC10861057; doi:10.1371/journal.pgen.1011146)
Supplement: S5 Table — (DOCX) [file pgen.1011146.s015.docx]

**Table S5. Oligonucleotides used in this study**

| Name | Target RNA | Probe (5'-3') | Sequence |
| --- | --- | --- | --- |
| LH 737 | tG(GCC) | 73-53 | TGCTTTGGCCGGGAATCGAAC |
| TDZ 126 | tP(AGG) | 72-53 | GGGCTGTTGTGGGAATCGAA |
| TDZ 128 | tY(GUA) | 73-52 | TCTCCTGAGCCAGAATCGAACT |
| TDZ 140 | tT(AGU) | 72-54 | GCTCCAGCAGTGACTCGAA |
| TDZ 184 | tC(GCA) | 22-1 | CTAACCACTGAGCTATGACCCC |
| OMT 625 | tG(GCC) | 76-59 | TGGTGCTTTGGCCGGGAA |
| AH 480 | tL(AAG) | 51-46-e6-e1-45-38 | CACGAGCTTTCGCACTAGTG |
| AH 497 | tL(UAG) | e5-e1-45-29 | TTTCGCACTGGCGCCTAAAGCC |
| AH 481 | tL(UAA) | 49-46-e8-e1-45-38 | CGCGTTTTACAACAGCAGGG |
| OMT 530 | tF(GAA) | 58-37 | TCGAACCGATGACCAACAGATC |
| ONV 22 | tL(CAA) | 76-53 | TGGTGACCAGTGAGGGATTCGAAC |
| ONV 15 | tL(CAG) | 49-27 | GTAGACTAGCACCTGAAGCTAG |
| ONV 9 | tS(CGA) | e9-e1-45-31 | GGCAGAGCCCATTAGATTTCGAGT |
| ONV 10 | tS(UGA) | e9-e1-45-28 | GGCAAAGCCCATTAGATTTCAAGTCTA |
| ONV 21 | tS(GCU) | 76-55 | TGGCGACAACGGCAGGATTCGA |
